# Supplementary material for: WordCommentsAnalyzer: A windows software tool for qualitative research
Source: F1000Res. 2018 Sep 4;7:536. Originally published 2018 May 3. [Version 2] doi: 10.12688/f1000research.14819.2 (PMC6137411; doi:10.12688/f1000research.14819.2)
Supplement: Supplementary file 3 [file f1000research-7-17614-s0002.tgz › b2a79496-4ada-47c4-9682-a99ce867836d.zip!/Eight Iranian Health Journals/Yafteh _47.docx]

© Yafteh  . This work is licensed under CC BY 4.0 (https://creativecommons.org/licenses/by/4.0/).

Volume & Issue: دوره 9 - شماره 2

Title: بررسی الگوی مقاومت دارویی استافیلوکوک کواگولاز منفی در کودکان

http://yafte.lums.ac.ir/article-1-30-fa.html

مقدمه : استافیلوکوک ‌کواگولاز منفی شایعترین میکروارگانیزمی است که در کشت‌های خون یافت می‌شود. تشخیص دقیق و آنتی‌بیوگرام این باکتری ارزش زیادی در درمان سپتی‌سمی های ناشی از این باکتری ایفاء می‌کند. هدف از این مطالعه ارزیابی حساسیت و مقاومت استافیلوکوک کواگولاز منفی جدا شده از کشت‌های خون کودکان بود. مواد و روشها: 270 نمونه کشت خون مثبت استافیلوکوک‌ کواگولاز منفی در طی 2 سال (83-1382) در مرکز پزشکی کودکان با روش‌های استاندارد آزمایشگاهی انتخاب و برای ارزیابی الگوی مقاومت دارویی از تکنیک دیفیوژن آگار (کریبی ـ بوئر) استفاده شد. یافته ها: در این مطالعه 4/54% از بیماران مذکر و 6/45% مونث بودند. میانگین جمعیت مورد مطالعه 10 ماه و حداقل سن یک روز و حداکثر 10 سال بودند. از 270 مورد کشت خون مثبت استافیلوکوک کواگولاز منفی 213 مورد ( 9/78%) عفونت واقعی و تعداد 57 مورد (1/21%) آلودگی خارجی بود. نتایج حاصل از آنتی‌بیوگرام نشان داد که استافیلوکوک های کواگولاز منفی نسبت به پنی‌سیلین 1/98% ، آمپی‌سیلین 9/95%، سفالکسین 3/62%، سفتی‌زوکیسم 9/55%، سفتریاکسون 7/53% و وانکومایسین 6/2% مقاوم هستند. بحث و نتیجه گیری: الگوی حساسیت و مقاومت دارویی استافیلوکوک کواگولاز منفی غیرقابل پیش‌بینی و مقاومت چندگانه به آن شایع است. وانکومایسین داروی انتخابی درمان سپتی‌سمی می‌باشد زیرا فقط(6/2%) موارد به آن مقاوم بودند.

Title: بررسی عاقبت حاملگی در خانم های باردار دریافت کننده واکسن سرخجه در طی طرح سراسری واکسیناسیون سرخک ـ سرخجه در استان لرستان

http://yafte.lums.ac.ir/article-1-31-fa.html

مقدمه : هدف از این مطالعه بررسی سرانجام حاملگی در خانم های باردار دریافت کننده واکسن سرخجه ـ سرخک در طی طرح سراسری واکسیناسیون سرخک ـ سرخجه در استان لرستان در پاییز 83 می باشد. مواد و روش ها: در این مطالعه اپیدمیولوژیک توصیفی تمام خانم های باردار دریافت کننده واکسن سرخجه ـ سرخک در دوره قبل از لقاح و بعد از لقاح در طی طرح سراسری واکسیناسیون وارد مطالعه شدند. اطلاعاتی نظیر عاقبت حاملگی، سندرم سرخجه مادرزادی، سن حاملگی موقع تولد و وزن موقع تولد از طریق یک پرسشنامه و چک لیست جمع آوری گردید و سپس این اطلاعات مورد تجزیه و تحلیل آماری قرار گرفتند. یافته ها: از تعداد 92 خانم دریافت کننده واکسن در 88 نفر از آنها عاقبت حاملگی شناخته شد (96%). از این تعداد 73 نفر آنها (83%) 74 نوزاد زنده و سالم بدنیا آوردند ، 5 نفر (6%) سقط خودبخود داشتند و 10 نفر (11%) بارداری را به طور اختیاری خاتمه دادند. در 74 نفر نوزاد زنده، متوسط سن حاملگی هنگام تولد 1/2 ± 5/39 هفته و وزن موقع تولد 535 ± 3257 گرم بود. هیچ یک از این 74 نفر نوزاد زنده، ناهنجاری که به عنوان سندرم سرخجه مادرزادی تلقی شود، نداشتند. بحث و نتیجه گیری: مطالعه نشان می دهد که واکسیناسیون سرخجه در حاملگی در عاقبت حاملگی مؤثر نمی باشد و یا سندرم سرخجه مادرزادی در استفاده کنندگان آن دیده نمی شود. در صورت تزریق اتفاقی واکسن سرخجه در زنان حامله نباید دلیلی برای آنها جهت ختم حاملگی در نظر گرفته شود. ولی هنوز هم حاملگی یک کنتراندیکاسیون برای تزریق واکسن سرخجه می باشد. زیرا از نظر تئوری ریسک ایجاد سندرم سرخجه مادرزادی وجود دارد .

Title: مقایسه میزان رضایت جنسی و افسردگی بین زوجین بارور و نابارور مراجعه کننده به مرکز آموزشی، درمانی الزهرا (س) تبریز 1385

http://yafte.lums.ac.ir/article-1-32-fa.html

Satarzadeh N1, Bahrami N2, Ranjbar Koch-saraei F3, Ghojazadeh M4 1. Instructor, Department of nursing, Faculty of nursing and midwifery, Tabriz University of medical sciences 2. Instructor, Department of parastari, Faculty of nursing and midwifery,Ghazvin University of medical sciences 3. Assistant professor, Department of psychology medicine , Faculty of medicine , Tabriz University of medical sciences 4. Assistant professor, Department of physiology , Faculty of medicine , Tabriz University of medical sciences Abstract Background: Some studies show the depression, stress, low self-esteem, sexual dissatisfaction may be psychological outcomes of infertility. Depression and sexual satisfaction play a significant role in the life of infertile couples, their infertility treatment follow- up and in their hopefulness for the future. The present study was carried out to determine and comparing the depression and sexual satisfaction among infertile and fertile couples referring to Alzahra educational center in Tabriz. Materials and methods: This is a comparative- descriptive study in which depression and sexual satisfaction rate were studied and analyzed in 150 infertile versus 150 fertile couples. Sample selection was randomized. Data gathering was done by using questionnaire including 3 parts( Demographic characteristics of couples, Beck Depression Inventory and Larson standard questionnaire for determining sexual satisfaction). All data gathered for this study was analyzed by using descriptive statistically methods, mean difference tests and also relation test. Results: The BDI mean difference between infertile and fertile men and between infertile and fertile women was significant (p=0.011,t=2.54in men & p<0.005, t=3.68in women). But the difference between sexual satisfaction mean difference in infertile and fertile men and between infertile and fertile women was not statically significant (p=0.707, t=0.377 in men & p=0.370, t=0.898 in women). Mean difference in infertile women was higher than their husbands but sexual satisfaction score in men and women was not statistically significant. Also there was a negative correlation between sexual satisfaction and depression. Conclusion: Infertile couples are indigent to exact care and treatment about their psychological problems. Presence of expert counselors in infertility treatment teams for diagnosing and treating these patients before any delay, is quite necessary.

Title: نقش کلونیدین در کاهش خونریزی حین عمل جراحی رینوپلاستی

http://yafte.lums.ac.ir/article-1-33-fa.html

Ø مقدمه: خونریزی حین عمل یکی از مهمترین عوارض در طی انواعی از جراحیها از جمله جراحی ستون مهره ها، جراحی پلاستیک مخصوصاً رینوپلاستی است. جهت کاهش این مشکل، تکنیک ها و داروهای زیادی استفاده می شود. در این مطالعه ما اثر پیش داروی کلونیدین خوراکی در ایجاد یک محدودۀ عمل تا حد امکان عاری از خون در بیماران تحت رینو پلاستی را مورد بررسی قرار داده ایم. Ø مواد و روش ها: این مطالعه یک کارآزمایی بالینی تصادفی دو سویه کور است که روی 40 بیمار ASA-1 کاندید عمل جراحی رینوپلاستی انتخابی تحت بیهوشی عمومی انجام گردیده است. بیماران بصورت تصادفی به دو گروه تقسیم شدند. 20 بیمار فقط دیازپام خوراکی mg/kg 1/0 و 20 بیمار هم دیازپام mg/kg 1/0 و هم کلونیدینkg / gµ 3 بصورت خوراکی 90 دقیقه قبل از آمدن به اتاق عمل دریافت کردند. روش بیهوشی در هر دو گروه یکسان بود. خونریزی حین عمل با استفاده از یک سیستم نمره دهی 4 تایی از 1 معادل خونریزی شدید تا 4، معادل بدون خونریزی و نیز میزان خون موجود در ساکشن و تعداد گازهای کاملاً خونی شده ارزیابی شد. این اطلاعات در پرسشنامه ثبت داده ها گردآوری شدند. تجزیه وتحلیل آماری با استفاده از آزمون تی نمونه ای مستقل تفاوت آماری معنی داری بین دو گروه نشان می داد (001/0p<). Ø یافته ها: متوسط سن افراد مورد مطالعه 6 ±9/ 24 سال بود. متوسط تعداد گازهای خونی شده در گروهی که کلونیدین دریافت کرده بودند 9/0±9/1 ودر گروه دیگر 1/1 ±6/3 بود. متوسط حجم خون موجود در ساکشن در گروهی که کلونیدین دریافت کرده بودند 28 ±65 و در گروه دیگر 30± 125 میلی لیتر بود. اختلاف آماری معنی داری بین جنس و متوسط سن دو گروه وجود نداشت (05/0p<). Ø بحث و نتیجه گیری: پیش درمانی با کلونیدین خوراکی 3 میکروگرم به ازای هر کیلوگرم وزن بدن میزان خونریزی در رینو پلاستی را کاهش داده و رضایتمندی جراح را افزایش می دهد.

Title: بررسی ویژگیهای فردی، خانوادگی، روانی و سوء مصر ف مواد در بیماران بستری با خودکشی در بیمارستان شهدای عشایر سال 83

http://yafte.lums.ac.ir/article-1-34-fa.html

مقدمه : خودکشی یکی از معضلات اجتماعی است که موجب از بین رفتن نیروی فعال جامعه شده و یک مشکل بزرگ برای سلامتی تلقی می شود. خودکشی نهمین علت مرگ و میر در ایالت متحده آمریکا است و نزدیک به 85 مورد خودکشی در هر روز (تقریباٌ یک مورد خودکشی در هر 20 دقیقه) اتفاق می افتد، سوء مصرف مواد خود یک فوریت روانپزشکی و از متغیرهای مهم مرتبط با خودکشی است مواد و روش ها: در این مطالعه همه بیمارانی که از تاریخ تیرماه 83 لغایت دیماه 83 اقدام به خودکشی نموده و در بیمارستان شهدای عشایر بستری گردیده بودند بصورت سرشماری انتخاب و در مطالعه وارد شدند. یافته ها: نتایج تحقیق ارتباط معنی داری را بین سوء مصرف مواد و بیماریهای جسمی مزمن در بیماران اقدام به خودکشی نشان داد. از 67 بیمار اقدام به خودکشی که در این مطالعه مورد بررسی قرار گرفتند 2/55 % مرد و 8/44% زن بودند. بیشترین فراوانی (2/55%) در گروه سنی 26-19 سال بوده و اکثریت نمونه ها (3/37%) مجرد بودند. 8/47% آنها بیکار بودند. 1/79% از واحدهای مورد پژوهش ساکن شهر و 9/21% ساکن روستا بودند. 7/53 % از واحدهای مورد پژوهش تحصیلات زیر دیپلم داشتند. بحث و نتیجه گیری: این پژوهش با هدف اصلی تعیین فراوانی مشخص نمودن ویژگیهای فردی، روانی، خانوادگی و سوء مصرف مواد در موارد اقدام به خودکشی انجام شد. نتایج این مطالعه مبین اثر پیشگیری کننده تحصیلات در این استان می باشد. همچنین نتایج نشان داد که هر چه تعداد افراد خانواده بیماران، بیشتر باشد، میزان اقدام به خودکشی نیز افزایش می یابد. افزون بر این مشخص شد که در بین متأهلین صاحب فرزند، اکثریت موارد بین 1 تا 2 فرزند داشتند که ا لبته با توجه به تعداد کم نمونه ها، بررسی های بیشتری را طلب می کند.

Title: بررسی میزان شیوع انگلهای گوارشی در بیماران HIV مثبت و ایدزی شهرستان

http://yafte.lums.ac.ir/article-1-35-fa.html

Ø مقدمه : انگلهای گوارشی از مهمترین عوامل بیماریهای روده ای در افراد مبتلابه ویروس HIV هستند. این پاتوژنهایکی از علل اصلی مرگ و میر در این بیماران به شمار می روند. مطالعات اندکی در مورد شیوع این انگلها در افراد HIV مثبت و مبتلا به ایدز در ایران انجام شده است. بنابراین برای تعیین میزان شیوع این پاتوژنهای فرصت طلب در بیماران HIV مثبت و مبتلا به ایدز ساکن شهرستان خرم آباد مطالعه ای بر روی 306 نفر از این بیماران انجام گرفت. Ø مواد و روشها : پس از انتخاب نمونه ها پرسشنامه های طراحی شده، تکمیل گردید و از هر فرد 3 نمونه مدفوع گرفته شد. بر روی هر نمونه دو تکنیک گسترش مستقیم و تغلیظ فرمالین- اتر و دو روش رنگ آمیزی اسید فست اصلاح شده (کینیون) و تریکروم اصلاح شده انجام گرفت. در ضمن جهت توصیف و تحلیل آماری داده ها از جداول توزیع فراوانی و آزمون استقلال کای-دو و نیز آزمون مستقل t استفاده شد. یافته ها : پس از انجام آزمایشها مشخص شد که شیوع انگلهای گوارشی در بیماران HIV مثبت و مبتلا به ایدز شهرستان خرم آباد در سال 1385 بطور کلی 5/22 % بوده که این میزان در بیماران مبتلا به ایدز به وضوح بالاتر بوده است.در ضمن نشان داده شد که بین متغیرهای مرکزی نمونه گیری، گروه سنی، میزان تحصیلات، شغل، نوع علائم گوارشی، وضعیت ابتلا به HIV و ایدز و آلودگی به انگلهای گوارشی ارتباط معنی داری وجود دارد. لازم به ذکر است که میان متغیر وضعیت ابتلا به HIV و ایدز و نوع انگل گوارشی نیز ارتباط معنی داری وجود داشت. بحث و نتیجه گیری : شیوع نسبتاً بالای انگلهای گوارشی در بیماران HIV مثبت و مبتلا به ایدز شهرستان خرم آباد لزوم اجرای برنامه های پیشگیری، غربالگری، تشخیص به موقع و درمان بیماران را ایجاب می کند.

Title: تاثیر برنامه آموزش پیشگیری از رفتارهای پر خطر بر آگاهی و نگرش مربیان بهداشت مدارس ابتدایی خرم آباد

http://yafte.lums.ac.ir/article-1-36-fa.html

مقدمه: رفتارهای پر خطر مهمترین عامل به خطر افتادن سلامت جامعه اند. امروزه شیوع رفتارهای پرخطر بخصوص در نوجوانان و جوانان به یکی از مهمترین و گسترده ترین دل نگرانیهای جوامع بشری تبدیل شده است. علیرغم فعالیتهای پیگیرانه سه دهه گذشته رفتارهای مخاطره آمیز در سطح جهان دارای رشد تصاعدی بوده است. هزینه پزشکی و مراقبتی آن رو به افزایش است. حال آنکه پیشگیری، تنها راه مقابله تشخیص داده شده است.تغییر رفتارهای بهداشتی مردم مستلزم اطلاع و آگاهی آنهاست. در این میان مربیان بهداشتی میتوانند سهم بسیار مهمی در ارتقاء آگاهی سایرین در جهت کنترل و پیشگیری ازرفتارهای پرخطر در مدرسه،خانواده و جامعه داشته باشند. لذا میزان آگاهی و آموزش مستمر آنها حائز اهمیت است. در این راستا پژوهش فوق با هدف تعیین تاثیر برنامه آموزش پیشگیری از رفتارهای پر خطر بر آگاهی و نگرش مربیان بهداشت مدارس ابتدایی خرم آباد در سال 1384 انجام گرفت. مواد و روش ها : پژوهش فوق بصورت نیمه تجربی است. کلیه مربیان بهداشت مدارس شهر خرم آباد (50 نفر) مورد بررسی قرار گرفتند. ابزار گرد آوری داده ها پرسشنامه چند قسمتی شامل مشخصه های فردی و سوالات آگاهی و نگرش نسبت به رفتارهای پرخطر بود که بر اساس اطلاعات و منابع موجود در کتب و مقالات معتبر علمی تدوین و پس از باز نگری وانجام پیش مطالعه و پایش مجدد مورد استفاده قرار گرفت.ابزار مداخله در این پژوهش برنامه آموزش پیشگیری از رفتارهای پر خطر بود که بصورت کارگاه در دو جلسه برگزار شد. محتوای آموزشی بیشتر بر ارائه آگاهی و نگرش مثبت به افراد مبتلا و رفتارهای پیشگیرانه در جهت ایجاد و تثبیت رفتارهای بهداشتی تاکید داشت که توسط اساتید صاحب نظر در این رشته اجرا گردید. 2 ماه پس از اجرای برنامه آموزشی با انجام آزمون دوم، نمونه ها مورد مطالعه و ارزیابی قرار گرفتند. پس از جمع آوری اطلاعات امتیاز سوالات مشخص و از طیف لیکرت برای تعیین آگاهی و نگرش استفاده شد. آگاهی کلی بصورت ضعیف (18-0، متوسط (35-19)، خوب (55-36) تعیین گردید. اطلاعات در دو مرحله جمع آوری شد. جهت تجزیه و تحلیل داده ها از آزمون های تی مستقل،تی زوج و انالیز واریانس استفاده شد. یافته ها: 7 /78% نمونه هاقبل از آموزش رفتارهای پر خطر را میشناختند و تنها 4/3% آنها توانستند رفتار پر خطر را درست تعریف نمایند. منابع اطلاعات اکثریت نمو نه ها در مورد رفتارهای پرخطر متنوع شامل رادیو و تلویزیون و کتب و نشریات کسب نموده بودند. آزمون آماری t زوج بین میانگین نمره های قبل و بعد از آموزش وآزمون آماری ویلکاکسون در نگرش قبل و بعد اختلاف معنی داری(001/0 p< ) نشان داد. بحث و نتیجه گیری: نتایج نشان داد که برنامه آموزش بهداشت طراحی شده در ارتقاء سطح آگاهی وتغییر یا ایجاد نگرش مثبت درافراد مورد مطالعه موثر بوده است. لذابا توجه به مسئولیت مهم مربیان در تامین و حفظ سلامت کودکان و جامعه، افزایش آگاهی و آموزش مستمر آنها، گنجاندن مطالب آموزشی در خصوص کنترل و پیشگیری از رفتارهای پرخطر در مواد درسی و تشکیل یک کمیته مشورتی بهداشت در مدرسه ضروری بنظر میرسد.

Title: تاثیر موسیقی بر دلیریوم بعد از جراحی مفصل ران در سالمندان

http://yafte.lums.ac.ir/article-1-37-fa.html

مقدمه : معمولا میزان بروز دلیریوم بعد از جراحی مفصل ران در سالمندان بالاست و باعث عوارض متعدد، پیش آگهی بد وحتی مرگ و میر می شود. با توجه به عوارض مختلف داروها، موسیقی می تواند یک روش غیر داروئی بی خطر جهت پیشگیری از دلیریوم باشد. هدف این مطالعه تعیین تاثیر موسیقی بر دلیریوم بعد از جراحی مفصل ران در زنان سالمند بستری در بخش ارتوپدی بوده است. مواد و روشها: این پژوهش یک مطالعه نیمه تجربی می باشد. نمونه های این پژوهش را 100 نفر اززنان سالمند دارای شکستگی مفصل ران بیمارستان شهدا خرم آباد در مدت 4ماه تشکیل می داد. با توجه به معیارهای ورود و روش نمونه گیری تصادفی به دوگروه 50 نفری آزمون و گواه تخصیص داده شدند. ابزار جمع آوری اطلاعات شامل پرسشنامه دموگرافیک و آزمون مختصر وضعیت روانی بود. در این پژوهش ابتدا با آزمون مختصر روانی وضعیت شناختی بیماران بررسی گردیده و برای افراد گروه آزمون، یک روز قبل از عمل، موسیقی باخ پخش شد. پخش موسیقی با استفاده از ضبط صوت مجهز به گوشی، هر بار بیست دقیقه ، سه بار در روز به مدت پنج روز برای افراد گروه آزمون انجام شد. گروه کنترل فقط مراقبت های معمول بخش را دریافت می کردند. بعد از انجام مداخله، در طی مداخله روزی دوبار آزمون مختصر وضعیت روانی از بیماران به عمل می آمد و وضعیت شناختی هر دو گروه بوسیله این آزمون سنجیده شد. تجزیه و تحلیل داده ها با استفاده از آزمون t مستقل و زوجی ،آنالیز کوواریانس وآزمون رتبه علامت دار ویلکاکسون انجام گردید. یافته ها: نتایج نشان می داد که میانگین نمره کل شناختی قبل از مداخله در گروه آزمون 78/21 و بعد از مداخله94/23 بود. مقایسه میانگین نمره کل شناختی بعد از مداخله در دوگروه نشان می دهد که بین میانگین نمره کل شناختی بعد از مداخله در دوگروه اختلاف معنی داری وجود دارد (018/0= p ). این اختلاف حتی با تعدیل اثر نمره کل شناختی قبل از مداخله (001/0 p< ) و تعدیل اثر سن (001/0 p< ) نیز معنی دار بود. بحث و نتیجه گیری: با توجه به یافته های پژوهش می توان نتیجه گرفت که موسیقی می تواند یک روش موثر و بی خطر غیر داروئی و رفتاری جهت پیشگیری از دلیریوم بعد از جراحی مفصل ران در سالمندان باشد.

Title: فیبروز ایدیوپاتیک ریوی کودکان: گزارش 8 مورد

http://yafte.lums.ac.ir/article-1-38-fa.html

مقدمه: فیبروز ریوی ایدیوپاتیک یک بیماری مزمن و نادر و معمولاً کشنده با منشا نامعلوم است که بیشتر در بالغین رخ می دهد این بیماری در کودکان و شیرخواران بسیار نادر است. معرفی مورد: 8 کودک مبتلا به فیبروز ریوی ایدیوپاتیک که در بخش اطفال در طی سالهای1380-1383 بستری شده بودند، تحت بررسی قرار گرفتند. از این تعداد 5 مورد پسر و 3 مورد دختر بوده که در سنین بین 7 تا 13 سالگی قرار داشتند (میانگین سنی 2/10 سال). علائم کلینیکی در کلیه بیماران بصورت سرفه و تنگی نفس بوده که سیر پیشرونده داشته و با عفونت های تنفسی مکرر تشدید یافته است. در معاینات بالینی رال های منتشر در هر دو ریه در تمام بیماران و سیانوز و کلابینگ در نزد 5 بیمار وجود داشته است. نمای رادیوگرافی سینه در 6 مورد بصورت رتیکولرندولر و در 2 مورد نمای بولوکیستیک بوده است. تستهای فونکسیون ریه در تمام بیماران منطبق با نمای بیماریهای تحدیدی ریوی می باشد. جهت تشخیص قطعی در کلیه بیماران بیوپسی ریه انجام شده که تغییرات بافت شناسی منطبق با فیبروز ریوی ایدیوپاتیک را مشخص کرده است. درمان بیماران عموماً با استروئید بوده است. در این مطالعه پیش آگهی بیماری تاکنون خوب بوده و فقط یک مورد مرگ بدنبال نارسائی بوده است. بحث و نتیجه گیری : بنظر می رسد که این مقاله گزارش اولین مورد فیبروز ریوی ایدیوپاتیک کودکان در ایران می باشد. مسلماً موارد متعدد این بیماری ممکن است در مراکز دیگر وجود داشته و لازم است با مشارکت محققین این موارد گرد آوری و مورد ارزیابی قرار گیرد.

Title: گزارش یک مورد سندرم باردت بیدل

http://yafte.lums.ac.ir/article-1-39-fa.html

مقدمه: سندرم باردت بیدل یک ناهنجاری ناهمگن با توارث اتوزومای مغلوب است. نشانه های اصلی بیماری شامل پیگمانتاسیون شبکیه، چاقی، پلی داکتیلی، عقب ماندگی ذهنی،بیماریهای کلیوی و هیپوگنادیسم است. نقص عملکرد کلیه علت اساسی مرگ هموزیگوت های مبتلاست، همراه با گلومرولوپاتی مزمن و پایین تر بودن مجرای ادرار که شکل های پیچیده و مبهم کلیوی را ایجاد می کند. ناهنجاری های ثانویه عبارت از اختلالات گفتاری، پیشرفت عقب ماندگی ذهنی، پر ادراری، دیابت ملیتوس و فشار خون بالا می باشند. شناسایی سندرم باردت- بیدل با یافته های کلینیکی صورت میپذیرد. دوازده ژن در ارتباط با سندرم باردت، بیدل شناسایی شده است که عبارتند از BBS1 و BBS2 و تا BBS12 . گزارش مورد: در این مقاله یک پسر چهارونیم ساله که حاصل یک ازدواج خویشاوندی درجه سوم و مبتلا به سندرم باردت بیدل می باشد معرفی می گردد. بحث و نتیجه گیری: سندرم تک ژنی مانند باردت بیدل دارای نشانه های فراوانی در فرد می باشد که نمایانگر پیامدهای پاتولوژیکی و کلینیکی حاصل از جهش در یک جایگاه تک ژنی است . بنابراین توصیه می شود افرادی که مایل به ازدواج فامیلی هستند پیش از ازدواج به مراکز مشاوره ژنتیک مراجعه نمایند تا شجره فامیلی آنان مورد بررسی قرار گرفته و ریسک ابتلا به سندرم باردت بیدل مشخص گردد.
